# Maternal satisfaction with intrapartum care and associated factors among mothers who gave birth in public hospitals of the South West Shewa Zone, Ethiopia, 2022

**DOI:** 10.3389/fgwh.2023.1203798

**Published:** 2023-10-03

**Authors:** Fikadu Tolesa, Adugna Alemu, Moges Beya, Mulugeta Feyisa, Andualem Gezahagn, Abdi Negash, Erean Shigign, Asfaw Getaye, Abraham Negash, Bacha Merga

**Affiliations:** ^1^ Department of Midwifery, Salale University, College of Health Sciences, Fitche, Ethiopia; ^2^ Department of Medical Laboratory, Salale University, College of Health Sciences, Fitche, Ethiopia; ^3^ Department of Public Health, Salale University, College of Health Sciences, Fitche, Ethiopia; ^4^ Department of Nursing, Salale University, College of Health Sciences, Fitche, Ethiopia; ^5^ School of Nursing and Midwifery, College of Health and Medical Sciences, Haramaya University, Harar, Ethiopia; ^6^ Ameya Hospital, South West Shewa Zone, Waliso, Ethiopia

**Keywords:** maternal satisfaction, intrapartum care, public hospitals, South West Shewa Zone, Ethiopia

## Abstract

**Background:**

Maternal satisfaction with intrapartum care is a multidimensional assumption of satisfaction with self and with the physical environment of the delivery ward and quality of care. Maternal satisfaction with intrapartum care affects the selection of birthplace and helps to identify gaps between actual and intended healthcare outcomes. This study aims to assess factors that affect maternal satisfaction with intrapartum care.

**Objectives:**

To assess maternal satisfaction with intrapartum care and associated factors among mothers who gave birth in public hospitals in the South-west Shewa Zone, Ethiopia, 2022.

**Methods:**

A cross-sectional study approach among 420 mothers was conducted between April 14 and June 14, 2022. Systematic random sampling was used to select mothers for face-to-face interviews every two intervals. Bivariate and multivariable logistic regression analyses were carried out. *P*-values of <0.25 in association with study variables were transferred to multivariable logistic regression models. An adjusted odds ratio with a 95% confidence interval was computed, and *p*-values of <0.05 were considered statistically significant in the multivariable model. The results of this study are presented using text, tables, and charts.

**Results:**

Data were collected from 420 participants, and 413 mothers completed the interview, giving a response rate of 98.33%. The overall maternal satisfaction with intrapartum care was 245 (59.32%) [95% CI: 55–64]. Mothers who were considered normal during labor and delivery (AOR = 2.57 (95% CI: 1.30–5.07), had a labor duration of 12 h or less (AOR = 1.59 (95% CI: 1.03–2.44), and experienced a waiting time of <15 min (AOR = 2.06 (95% CI: 1.21–3.52) were significantly associated with maternal satisfaction with intrapartum care.

**Conclusion and Recommendations:**

More than half of mothers were satisfied with the overall intrapartum care they received. Health facility managers and healthcare providers work together to improve maternal satisfaction with intrapartum care.

## 1. Introduction

Satisfaction is difficult to define conceptually because it is affected by a variety of factors. Expectations of mothers, which might vary across women due to different contexts, such as their socioeconomic background, education level, and individual preferences, are among the various elements that determine this level of satisfaction (1). Maternal satisfaction refers to a mother's feelings of joy as a result of comparing a service provided in a health facility to her expectations and the ability of the services provided to meet her expectations, which is a crucial factor when choosing a health facility, compliance with services and follow-ups, and continuation of healthcare (2).

Users' satisfaction with intrapartum care is a comprehensive concept that involves one's contentment, the physical environment of the delivery ward, and the degree of care delivered. Women's satisfaction during childbirth is the most widely reported criterion for evaluating the quality of childbearing services (3).

The World Health Organization (WHO) defines intrapartum care as a platform for providing pregnant women with respectful, individualized, woman-centered, and effective clinical and non-clinical practices to improve birth outcomes for mother and baby by having skilled healthcare providers provide intrapartum care in a well-functioning healthcare system. Women seek a “good childbirth experience” that meets or exceeds their past personal and societal beliefs and expectations according to the evidence used to establish the 2018 WHO recommendations on intrapartum care (4).

Physical environment and infrastructure, continuity of service provided to mothers, access, information, cost, and attention to psychological issues are all factors that can influence maternal satisfaction (3, 5). Mothers' satisfaction is an outcome indicator of the quality and efficiency of care services in the healthcare system, professional healthcare being an important indicator of overall client satisfaction (6).

Maternal satisfaction with intrapartum care is closely tied to service utilization and the perception of care outcomes that fulfill mothers' expectations (3). Globally in 2017, more than 830 women died within 24 h from cases related to pregnancy and childbirth process problems, and 94% of all maternal mortality occurs in low and middle-income countries (7). As part of Sustainable Development Goal 3, countries have committed to decreasing the maternal mortality ratio to less than 70 per 100,000 live births between 2015 and 2030. Therefore, addressing current levels of maternal and neonatal mortality in low- and middle-income countries by 2030 is a global priority (8). The burden of maternal and neonatal mortality is highest in Sub-Saharan Africa, where estimates of severe maternal morbidity range up to 108 per 1,000 live births (9). Maternal mortality is high at-home deliveries (10). Utilization of healthcare services and the belief that the care provided meets clients' expectations are directly related to women's satisfaction (11).

Studies carried out in Malaysia and Nepal revealed that 79.2% and 89.88% of women were satisfied with the services received during childbirth respectively (11, 12). Maternal satisfaction among women who gave birth in African nations was 78.54%, 94%, and 92.54% in Egypt, Ghana, and Mozambique, respectively (13–15). However, in Ethiopia, the proportion of women who were satisfied with child bearing care ranged from 19% to 87.2% (16–21).

Long waiting times, improper availability of drugs and supplies, disrespect, lack of privacy, poor cleanliness in health facilities, poor communication, unprofessional healthcare behavior, unplanned pregnancy, and complicated feto-maternal birth outcomes were factors that had negative impacts on maternal satisfaction with intrapartum care (2, 15, 22–25).

Mothers who denied the friendly approaching behavior of the healthcare provider during childbirth preferred traditional birth attendance (26, 27). Women who were dissatisfied with the quality of care during labor and delivery preferred to give birth at home in the future (28). The dissatisfaction of mothers with childbirth services was an important barrier to women seeking institutional delivery (29, 30). Maternal satisfaction with childbirth provides crucial and cost-effective feedback for further improving institutional childbearing services (11). Satisfaction with intrapartum care has long-term and immediate benefits for the health of the mother and the subsequent uptake and recommendation of the institution's care among their neighbors and relatives (6).

Ethiopia has made remarkable progress in expanding healthcare services through the rapid expansion of infrastructure, increased availability of healthcare providers, increased budget allocation, and enhanced financial management. However, only half of all women give birth in a hospital. Until now, maintaining and improving the quality of service has been a major challenge. As a result, providing high-quality healthcare has become a major transformation priority (31). An Ethiopian mini demographic survey showed that only 48% of live births took place at a health facility (32).

Small studies on maternal intrapartum care satisfaction have been conducted in Ethiopia, and the levels of maternal satisfaction with intrapartum care vary from region to region. Furthermore, there was no study conducted on maternal satisfaction with intrapartum care in the South West Shewa zone. The results of this study will help healthcare providers, hospital managers, local planners and decision-makers, and other stakeholders better understand how well services are provided, how well providers have met clients' expectations, and what changes may be required to meet clients' expectations and increase service utilization, which has a significant positive impact on mothers' and newborns' lives. Therefore, this study aims to assess factors associated with maternal satisfaction with intrapartum care in public hospitals in the South West Shewa Zone, Ethiopia.

## 2. Methods and materials

### 2.1. Study area and study period

The study was carried out in public hospitals in the South West Shewa Zone, Oromia region, Ethiopia. Based on the 2007 census conducted by the Central Statistical Agency of Ethiopia, this zone has a total population of 1,101,129, of whom, 556,194 are men and 544,935 are women (33). Southwest Shewa Zone consists of six hospitals (five government hospitals and one non-government hospital), 54 health centers, 264 health posts, 72 private clinics, eight private pharmacies, and 28 drug stores. The study was conducted from April 14–June 14, 2022.

### 2.2. Study design

A facility-based, cross-sectional study was conducted.

### 2.3. Study population

All selected mothers who gave birth in public hospitals in the South West Shewa Zone from April 14–June 14, 2022.

### 2.4. Inclusion criteria and exclusion criteria

Mothers who gave birth and were discharged from hospital by healthcare providers were included in the study. However, mothers who gave birth to stillborn babies or who suffered serious health issues and were unable to communicate during face-to-face interviews were not included.

### 2.5. Sample size and sampling procedure

The sample size was calculated using a single proportion population formula for the first objective, with the assumption of 95% confidence interval, 5% margin of error, 45% maternal intrapartum satisfaction), based on a previous study conducted in Public Health Facilities, Jimma Zone (34) and 10% of non-response rate. The final sample size for the study was found to be 420. The sample size for the second objective was also calculated using a double population proportion formula in Epi Info version 7.2.5; however, this is less than the first objective sample size.

**Figure 1:**
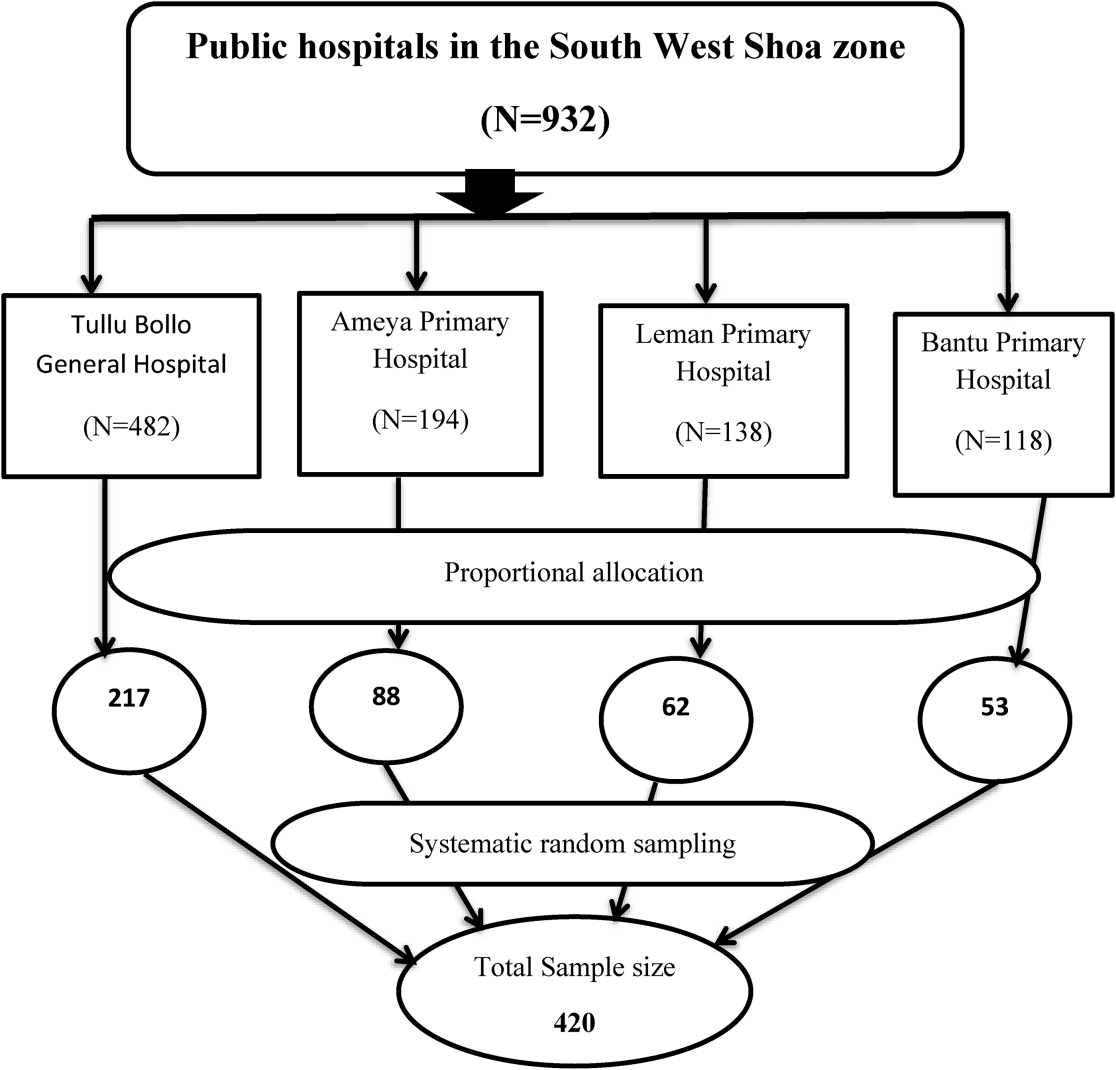
Schematic presentation of sampling procedure on maternal satisfaction with intrapartum care and associated factors among mothers who gave birth in public hospitals in the South West Shewa Zone, Oromia region, Ethiopia, 2022.

The study participants were selected using a systematic random sampling technique, and the sampling interval was determined by dividing the number of average monthly delivered mothers by their allocated sample size at each hospital, which is K = N/*n *∼ 2. Therefore, every other two postpartum women was included in the study until the required allocated sample at each hospital was achieved at the time the healthcare provider decided to discharge, but before leaving the hospital (See Figure 1).

### 2.6. Data collection tools and quality control

Data were collected by pretested structured face-to-face interviewer-administered questionnaire adapted from related literature (2, 12, 19, 20, 24, 37, 38). To ensure the consistency of the questionnaires, the English version was translated into Afaan Oromo for data collection and retranslated into English by a professional translator. The questionnaires contained three parts and 45 items composed of 7 items relating to socio-demographic characteristics, 10 items relating to obstetrics-related factors, and 28 items relating to satisfaction assessment questions.

Pretesting was conducted on 50 study participants (50 mothers) who gave birth in Inchini Primary Hospital. The collected data were entered into EpiData version 4.62 and then exported to SPSS version 25. Cronbach's alpha of the tools was assessed, which was 0.85. One day of training was provided for the data collectors and supervisors regarding the data collection procedure, tools, the purpose of the study, and ethical considerations. Data collectors were strictly supervised. At the end of each day, the questionnaire was reviewed and checked for completeness by the supervisors, and corrections were made.

### 2.7. Variables

The dependent variable was maternal satisfaction with intrapartum care and the independent variables were socio-demographic characteristics (age, place of residence, educational status, occupational status) and obstetric history of mothers (parity, duration of labor, status of current pregnancy, mode of delivery, maternal outcome, fetal outcome, ANC follow-up, waiting time).

### 2.8. Operational definitions

Maternal satisfaction with intrapartum care was measured using 28 items, composed of three dimensions: interpersonal care aspect (14 items), information aspect (7 items), and physical birth environment (structural) (7 items). Participants were asked to rate their satisfaction level using a 5-point Likert scale (1 = strongly disagree, 2 = disagree, 3 = neutral, 4 = agree, and 5 = strongly agree). The 5-point Likert scale was merged into two categories of outcome variables for analysis purposes. Strongly disagree, disagree, and neutral responses were coded as “0”, whereas agree and strongly agree were coded as “1”. Then, responses to 28 measuring items were added and converted to give an overall score for the women's levels of satisfaction.

**Satisfied:** Mothers who scored 75% or more in the 28 items of the mothers’ satisfaction questionnaires were categorized as satisfied with the overall care received.

**Unsatisfied:** Mothers who scored below 75% in the 28 items of the mothers' satisfaction questionnaires were categorized as unsatisfied for overall satisfaction (17, 34, 37, 39, 40).

**Waiting time**: The time between admissions to the time seen by the healthcare providers (41).

**Maternal outcome:** Mothers were considered to have had complications if they developed at least one of the following: postpartum hemorrhage, puerperal sepsis, perineal and cervical lacerations, and retained placenta. Mothers who had none of the complications listed above and were clinically assessed as good were considered normal.

### 2.9. Data processing and analysis

The collected data were checked for completeness, entered into the EpiData manager version 4.62, and exported to SPSS version 25 for data analysis. Descriptive analysis (frequencies, percentages, means, and standard deviation) and inferential analysis were conducted. Variance inflation factors (VIF) were utilized to screen for multicollinearity and a VIF of less than 10 was employed as a cutoff point to diagnose multicollinearity.

Model goodness of fit was checked by the Hosmer–Lermeshow test and was found to be 0.78. Bivariate and multivariable logistic regression analyses were conducted. Variables with a *p*-values 0.25 in bivariate analysis were transferred to multivariable analysis. AOR (adjusted odds ratio) with a 95% confidence interval was computed, and the *p*-value < 0.05 was considered statistically significant in the multivariable. The results are presented using text, tables, and charts.

### 2.10. Ethical consideration

Ethical clearance to conduct the study was obtained from the Salale University College of Health Science Ethical Review committee (Ref number HSC/878/2022). Informed written consent was also obtained from each study participant after the objectives of the study were explained. Participation in the study was based on their volunteer status. The information obtained from the participants was kept confidential. All methods were carried out in accordance with relevant guidelines and regulations.

## 3. Results

A total of 420 women were interviewed, making the response rate 98.33%. The seven questionnaires that were not completed were excluded from the analysis (Table 1).

**Table 1:** Sample size determination for the second objective of intrapartum care maternal satisfaction among mothers who gave birth in the South West Shewa Zone, Oromia region, Ethiopia, 2022.

Variables	CI	Power	P1	P2	AOR	Ratio	Sample size	10% Non-response rate	Final sample size	Reference
	95%	80%								
Duration of labor	95%	80%	69.6	10.1	3.03	1:1	220	22	242	(35)
Residency	95%	80%	49.5	10.4	2.63	1:1	290	29	319	(36)
Maternal outcome	95%	80%	67.2	16.7	3.597	1:1	116	12	128	(37)
Parity	95%	80%	42.7	47.2	2.352	1:1	198	20	218	(12)

P1, Proportion of outcome among population with the exposure of interest. P2, Proportion of outcome among population without the exposure of interest.

### 3.1. Socio-demographic characteristics of the respondents

The minimum age of respondents was 18 years, while the maximum was 45 years, with a mean age of 28.04 (SD± 6.63). Among the 413 study participants, more than half, (236, 57.1%) of women were living in rural areas. More than three-quarters of the study participants were married (314, 76%), and 141 (34.1%) women had completed secondary education (see Table 2).

**Table 2:** Socio-demographic characteristics of mothers who gave birth in public hospitals, South West Shewa, Ethiopia, 2022 (*n* = 413).

Variables	Variables categories	Frequency	Percentage
Age	≤24	145	35.1
	25–29	101	24.5
	30–34	89	21.5
	≥35	78	18.9
Residency	Urban	177	42.9
	Rural	236	57.1
Marital status	Married	314	76
	Unmarried	99	24
Educational status	No formal education	83	20.1
	Primary Education	128	31
	Secondary Education	141	34.1
	Diploma and above	61	14.8
Occupational status	Housewife	101	24.5
	Farmer	93	22.5
	Merchant	91	22
	Government employee	57	13.8
	Non-government employee	71	17.2
Reason for going to the hospital	By referral	175	42.4
	By friends and relatives	77	18.6
	By self	161	39
Type of visit	New	263	63.7
	Repeat	150	36.3

### 3.2. Maternal obstetrics characteristics

In this study, among the 413 respondents, more than half (237, 57.4%) of mothers were multiparous. More than three-quarters (323, 78.2%) of women had planned their current pregnancy. The majority of the respondents (354, 85.7%) had ANC follow-ups. However, only 171 (48.3%) women completed the fourth visit (see Table 3).

**Table 3:** Obstetrics characteristics of mothers who gave birth in public hospitals, South West Shewa, Ethiopia, 2022 (*n *= 413).

Variables	Variables categories	Frequency	Percentage
Parity	Primiparous	176	42.6
	Multiparous	237	57.4
Status of current pregnancy	Planned	323	78.2
	Unplanned	90	21.8
ANC follow-up	Yes	354	85.7
	No	59	14.3
Number of ANC visit (*n* = 354)	First visit	12	3.4
	Second visit	48	13.6
	Third visit	123	34.7
	Fourth visit	171	48.3
Labor duration	Less than or equal to 12 h	209	50.6
	Greater than 12 h	204	49.4
Mode of delivery	Spontaneous vaginal delivery	218	52.8
	Instrumental delivery	81	19.6
	Cesarean section	114	27.6
Maternal outcome	Normal	365	88.4
	With complications	48	11.6
Mothers with complications (*n* = 48)	Postpartum hemorrhage	20	41.7
	Puerperal sepsis	9	18.8
	Perineal and cervical laceration	14	29.1
	Retained placenta	5	10.4
Fetal outcome	Normal	305	73.8
	With complication	108	26.2
Waiting time	<15 min	216	52.3
	15–30 min	93	22.5
	>30 min	104	25.2

### 3.3. Maternal satisfaction with intrapartum care

**Figure 2:**
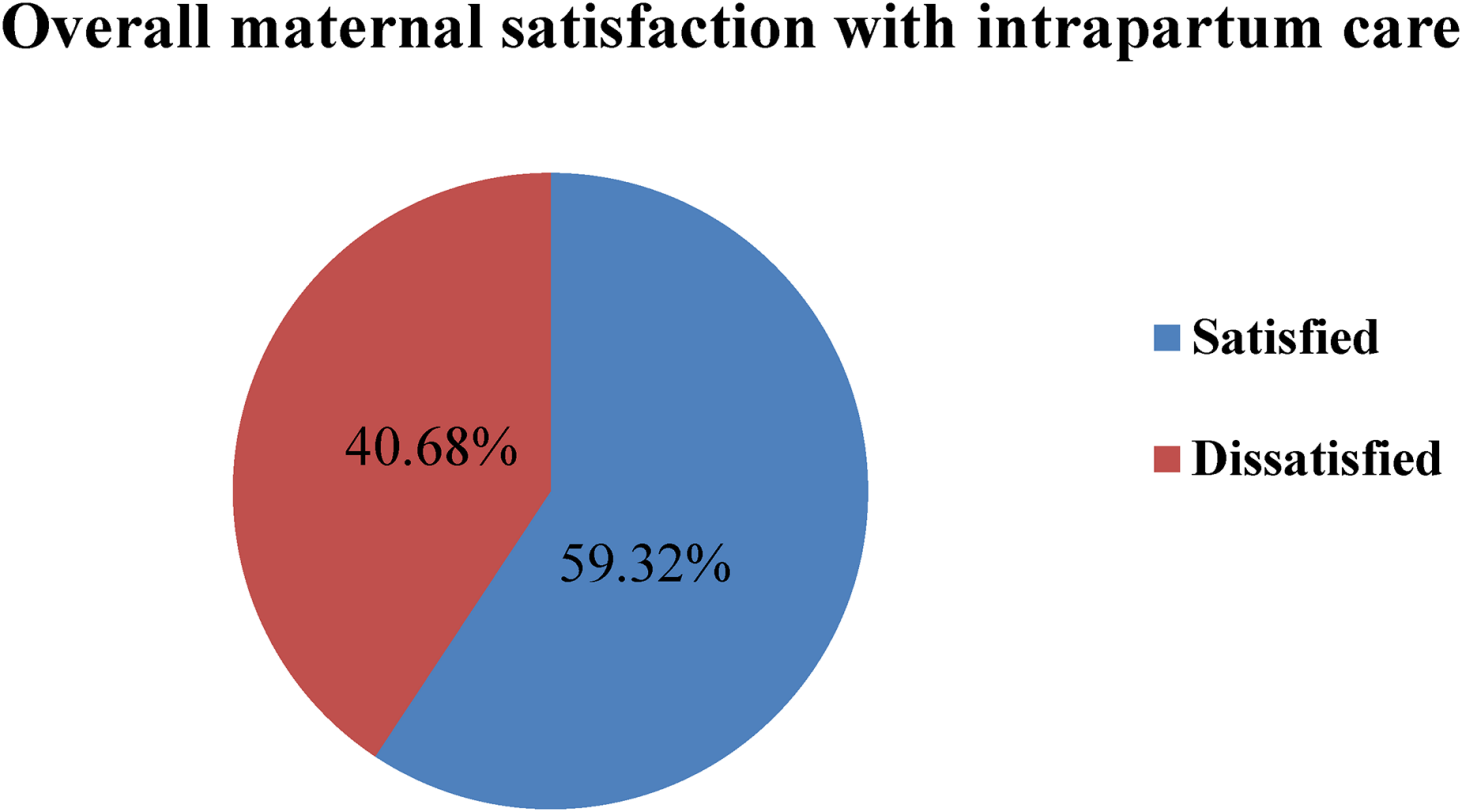
Pie chart shows the overall maternal satisfaction with intrapartum care among mothers who gave birth in public hospitals in the South West Shewa Zone, Ethiopia, 2022.

The findings of this study reveal that the overall maternal satisfaction with intrapartum care was 245 (59.32%) [95% CI: 55–64] (see Figure 2).

### 3.4. Maternal satisfaction with intrapartum care related to interpersonal care

The findings of this study show that 268 (64.9%) of the respondents were satisfied with the privacy they were provided during childbearing. Among 413 mothers, more than one-third (295, 71.4%) received a warm welcome. Regarding healthcare providers' stay with mothers, 315 (76.3%) of the mothers were satisfied. More than three-quarters (332, 80.4%) of women were satisfied with the monitoring of their labor progress by caregivers. Concerning assistance in early ambulation, 318 (77%) mothers were satisfied with the intrapartum care they received (see Table 4).

**Table 4:** Interpersonal-related maternal satisfaction with intrapartum care among mothers who gave birth in public hospitals in the South West Shewa Zone, Ethiopia, 2022 (*n *= 413).

Variables	Variables category	Frequency	Percentage
Available doctors and midwives	Satisfied	341	82.6
	Dissatisfied	72	17.4
Respecting mother while giving care	Satisfied	283	68.5
	Dissatisfied	130	31.5
Examination with mother's permission	Satisfied	332	80.4
	Dissatisfied	81	19.6
Assured privacy	Satisfied	268	64.9
	Dissatisfied	145	35.1
Received by welcoming	Satisfied	295	71.4
	Dissatisfied	118	28.6
Listening and answering all the mother's questions	Satisfied	318	77
	Dissatisfied	95	23
Labor pain management	Satisfied	314	76
	Dissatisfied	99	24
Allowing families to stay with laboring mothers	Satisfied	317	76.8
	Dissatisfied	96	23.2
Monitoring FHB	Satisfied	332	80.4
	Dissatisfied	81	19.6
Monitoring progress of labor	Satisfied	332	80.4
	Dissatisfied	81	19.6
Healthcare providers stay with mothers	Satisfied	315	76.3
	Dissatisfied	98	23.7
Keeping mother in a comfortable position	Satisfied	278	67.3
	Dissatisfied	135	32.7
Assisting in perineal care	Satisfied	290	70.2
	Dissatisfied	123	29.8
Assisting in early ambulation	Satisfied	318	77
	Dissatisfied	95	23

### 3.5. Maternal satisfaction with intrapartum care related to information

According to the result of the current study, 252 (61%) mothers were satisfied with the information given about the result of their examination. A total of 323 (78.2%) and 267 (64.6%) of the respondents were satisfied with the information given by healthcare providers about the progress of their labor and personal hygiene, respectively (see Table 5).

**Table 5:** Information-related maternal satisfaction with intrapartum care among those who gave birth in public hospitals in the South West Shewa Zone, Ethiopia, 2022(*n *= 413).

Variables	Variables category	Frequency	Percentage
Information about the results of an examination	Satisfied	252	61.0
	Dissatisfied	161	39.0
Information about the progress of labor	Satisfied	323	78.2
	Dissatisfied	90	21.8
Information about the outcome of newborn	Satisfied	317	76.8
	Dissatisfied	96	23.2
Information about breastfeeding	Satisfied	318	77
	Dissatisfied	95	23
Information about personal hygiene	Satisfied	267	64.6
	Dissatisfied	146	35.4
Information about newborn care and immunization	Satisfied	267	64.6
	Dissatisfied	146	35.4
Consent requested before the procedure	Satisfied	335	81.1
	Dissatisfied	78	18.9

### 3.6. Maternal satisfaction with intrapartum care related to health facility structure

The results of this study showed that 321 (77.7%) of the respondents were satisfied with the cleanliness of the delivery room. Among the 413 mothers, 331 (80.1%) and 330 (79.9%)were satisfied with getting prescribed drugs and laboratory tests in the health facilities, respectively. Concerning the availability and cleanliness of toilets, less than half (203, 49.2%) of the mothers were satisfied (see Table 6).

**Table 6:** Structure-related maternal satisfaction with intrapartum care among mothers who gave birth in public hospitals in the South West Shewa Zone, Ethiopia, 2022 (*n *= 413).

Variables	Variable category	Frequency	Percentage
Cleanliness of delivery room	Satisfied	321	77.7
	Dissatisfied	92	22.3
Comfort of the waiting area	Satisfied	315	76.3
	Dissatisfied	98	23.7
Getting prescribed drugs and supplies	Satisfied	331	80.1
	Dissatisfied	82	19.9
Getting laboratory tests	Satisfied	330	79.9
	Dissatisfied	83	20.1
Cleanliness of bed	Satisfied	325	78.7
	Dissatisfied	88	21.3
Availability and cleanliness of bathroom	Satisfied	217	52.5
	Dissatisfied	196	47.5
Availability and cleanliness of toilet	Satisfied	203	49.2
	Dissatisfied	210	50.8

### 3.7. Factors associated with maternal satisfaction with intrapartum care

According to this study, the age of mothers, marital status, educational status, labor duration, maternal complications during labor and delivery, fetal outcome, and waiting time were identified as candidate variables for multivariable logistic regression analysis. After controlling for possible confounding variables by multivariable logistic analysis, labor duration, maternal complications during labor and delivery, and waiting time were significantly associated with maternal satisfaction with intrapartum care at a *p*-value of <0.05.

This study's findings show that the mothers whose labors lasted 12 h or less were [AOR = 1.59 (95% CI:1.03–2.44)] more likely to be satisfied with their intrapartum care than those whose labors lasted longer than 12 h. In this study, women who did not develop obstetric complications during labor and delivery were [AOR = 2.57(95% CI: 1.30–5.07)] more likely to be satisfied compared to those who developed obstetric complications. In addition, women who waited 15 min or less to be seen by the healthcare providers were [AOR = 2.06 (95% CI: 1.21–3.52)] two times more likely to be satisfied compared to women who waited more than 30 min (see Table 7).

**Table 7:** Bivariate and multivariable logistics regression analysis for maternal satisfaction with intrapartum care among mothers who gave birth in public hospitals in South West Shewa, Ethiopia, 2022 (*n *= 413).

Variables	Variables category	Satisfaction		*P*-value	COR 95% CI	*P*-value	AOR 95% CI
		Satisfied, *N* (%)	Dissatisfied, *N* (%)				
Age	≤24	95 (65.5%)	50 (34.5%)	0.015	2.00 (1.14–3.50)	0.343	1.38 (0.71–2.71)
	25–29	58 (57.4%)	43 (42.6%)	0.247	1.42 (0.78–2.57)	0.814	1.09 (0.55–2.16)
	30–34	54 (60.7%)	35 (39.3%)	0.122	1.62 (0.88–3.00)	0.448	1.30 (0.66–2.58)
	≥35	38 (48.7%)	40 (51.3%)		1		
Marital status	Unmarried	50 (50.5%)	49 (49.5%)		1	0.065	0.62 (0.37–1.03)
	Married	195 (62.1%)	119 (37.9%)	0.041	1.61 (1.02–2.53)		
Educational status	No formal education	49 (59.0%)	34 (41.0%)	0.080	1.81 (0.93–3.54)	0.247	1.55 (0.74–3.27)
	Primary Education	82 (64.1%)	46 (35.9%)	0.011	2.24 (1.22–4.17)	0.129	1.72 (0.85–3.46)
	Secondary Education	87 (61.7%)	54 (38.3%)	0.023	2.03 (1.10–3.73)	0.147	1.66 (0.84–3.27)
	Diploma and above	27 (44.3%)	34 (55.7%)		1		1
Labor duration	≤12 h	135 (64.6%)	74 (35.4%)	0.028	1.56 (1.05–2.31)	**0**.036*	**1.59** (**1.03–2.44)**
	>12 h	110 (53.9%)	94 (46.1%)		1		
Maternal outcome	Normal	229 (62.7%)	136 (37.3%)	0.001	3.37 (1.78–6.36)	**0**.006*	**2.57** (**1.30–5.07)**
	With complications	16 (33.3%)	32 (66.7%)		1		1
Fetal outcome	Normal	199 (65.2%)	106 (4.8%)	0.001	2.53 (1.62–3.96)	0.067	1.60 (0.97–2.65)
	With complications	46 (42.6%)	62 (57.4%)		1		1
Waiting time	<15 min	145 (67.1%)	71 (32.9%)	0.001	2.78 (1.72–4.51)	**0**.008*	**2.06** (**1.21–3.52)**
	15–30 min	56 (60.2%)	37 (39.8%)	0.013	2.06 (1.17–3.65)	0.097	1.67(0.91–3.06)
	>30 min	44(42.3%)	60(57.7%)		1		1

* = statistically significant, 1 = Reference. COR, Crude odd ratio; AOR, Adjusted odd ratio; CI, confidence interval; N, frequency.

## 4. Discussion

Maternal satisfaction with labor and delivery service is an important outcome measure for the quality of care and provision of services. This study aims to assess maternal satisfaction with intrapartum care at public hospitals in South West Shewa. Accordingly, the overall percentage of mothers who were satisfied with intrapartum care in this study was 59.32% [95% CI: 55–64]. This study is in line with the study conducted in the West Shewa zone (60.8%) (40) and the Bench-Maji Zone, Ethiopia (63.25%) (36). The probable justification for the observed inline findings could be due to the study design and study setting because both studies were conducted using a facility-based cross-sectional study design at the zonal level.

However, the findings of this study are higher than the studies conducted in West Shewa, Central Ethiopia (36.6%) (42), at St Paul's Hospital Millennium Medical College, Addis Ababa Ethiopia (19%) (16), in Gondar Teaching Hospital, Northwest Ethiopia (31.3%) (43). The difference in the study area, the standard of the healthcare facility providing intrapartum care, and the variation in the quality of the care provided could all be contributing factors to this variance.

On the contrary, the findings of this study are lower than the studies conducted in Ethiopia, such as in West Gojjam (88%) (24), Hawassa City (87.7%) (17), Wolaita Sodo University Teaching and Referral Hospital (67.3%) (38), Harar hospitals, Eastern Ethiopia (84.7%) (44), Egypt (78.5%) (13). Variations in the research environment, health facility infrastructures, and socio-demographic characteristics of the study populations might be the cause of these disparities.

This study found that a mother who was considered normal after delivery was more likely to be satisfied with intrapartum care. This finding is similar to that of a study conducted in Nekemte Specialized Hospital in Western Ethiopia (2). This is due to the possibility that women without difficulties may be satisfied with the care they received, which could result in satisfaction. The other probable justification might be that mothers with complications blame the service of the health facility and give a negative response. Another reason could be mothers who experience complications during intrapartum may believe it to be the fault of the healthcare givers who attended her or the hospital in general, resulting in reduced trust in delivering at a health facility.

According to the current study, women whose labor lasts 12 h or less are more likely to be satisfied with their intrapartum care than mothers whose labor lasts more than 12 h. This was similar to the previous study conducted in Eastern Ethiopia (45) and Nekemte Specialized Hospital in Western Ethiopia (2). This could be a result of the fact that prolonged labor can exhaust a woman and subject her to repeated obstetric procedures like vaginal examinations (46), and as time goes on, anxiety about the birth's outcome may also rise, decreasing maternal satisfaction (47).

Furthermore, the findings of this study show that immediate care without delay as soon as women arrived at health facilities had a positive association with maternal satisfaction with intrapartum care. Women who waited less than 15 min to be seen by healthcare providers were more satisfied with their intrapartum care. This study was supported by a study conducted in the Harari regional state, Ethiopia (45) and Nekemte Specialized Hospital in Western Ethiopia (2). This might be a result of providing immediate care to women in labor, which could meet their expectations and lessen complications during labor and delivery, leading to maternal satisfaction.

## 5. Conclusion and recommendations

The results of the current study conclude that more than half of mothers were satisfied with their intrapartum care. Mothers' labor and delivery outcomes, labor duration, and waiting time were significantly associated with maternal satisfaction with intrapartum care.

Hospital managers and healthcare providers should focus their efforts on labor and delivery services in order to increase maternal satisfaction with intrapartum care. Additionally, in order to increase maternal satisfaction with intrapartum care, waiting times should be decreased, extended labor should be managed immediately, and problems during labor and delivery should be identified and treated early.

## 6. Limitations of the study

Limitations of this study include its cross-sectional design, which is unable to show a cause-and-effect relationship; its restriction to public hospitals; and potential response biases due to social desirability. However, by interviewing the women as they prepared to leave hospital, the authors aimed to reduce prejudices.

## Data Availability

The original contributions presented in the study are included in the article/Supplementary Material, further inquiries can be directed to the corresponding author.

## References

[1] Okonofua F Ogu R Agholor K Okike O Abdus-salam R Gana M Qualitative assessment of women ’ s satisfaction with maternal health care in referral hospitals in Nigeria. Reprod Health. (2017) 14:1–8. 10.1186/s12978-017-0305-6 28302182 PMC5356406

[2] Babure ZK Assefa JF Weldemarium TD. Maternal satisfaction and associated factors towards delivery service among mothers who gave birth at nekemte specialized hospital, nekemte town, east wollega zone, oromia regional state, western Ethiopia, 2019: a cross- sectional study design. J Women’s Health Care. (2019) 9:2167–0420. 10.35248/2167-0420.20.9.489.Copyright

[3] Mehata S Paudel YR Dariang M Aryal KK Paudel S Mehta R Factors determining satisfaction among facility-based maternity clients in Nepal. BMC Pregnancy Childbirth. (2017) 17:1–10. 10.1186/s12884-017-1532-0 28946851 PMC5613378

[4] Oladapo OT Tunçalp Ö Bonet M Lawrie TA Portela A Downe S WHO Model of intrapartum care for a positive childbirth experience: transforming care of women and babies for improved health and wellbeing. BJOG: Int J Obste Gynaecol. (2018) 125(8):918–22. 10.1111/1471-0528.15237 PMC6033015 29637727

[5] Lazzerini M Mariani I Semenzato C Valente EP. Association between maternal satisfaction and other indicators of quality of care at childbirth: a cross- ­ sectional study based on the WHO standards. BMJ Open. (2020) 10:e037063. 10.1136/bmjopen-2020-037063 32928854 PMC7490935

[6] Battawi JAA-Hafiz SK. Evaluation of postnatal mother ’ s satisfaction with nursing care in El-Shatby maternity university hospital. J Nurs Health Sci (IOSR-JNHS). (2017) 6(6):69–80. 10.9790/1959-0606026980

[7] WHO. Maternal mortality evidence brief. Geneve, Switzerland: World Health Organization (2017). 1–4.

[8] WHO and Group. ‘Trends in Maternal Mortality: 1990 to 2015’ (2015).

[9] Geller SE Koch AR Garland CE Jane MacDonald E Storey F Lawton B. A global view of severe maternal morbidity: moving beyond maternal mortality. Reprod Health. (2018) 15(Suppl 1):31–43. 10.1186/s12978-018-0527-2 29945657 PMC6019990

[10] Chernet AG Dumga KT Cherie KT. Home delivery practices and associated factors in Ethiopia. J Reprod Infertil. (2019) 20(2):102–8. 31058055 PMC6486567

[11] Jha P Larsson M Christensson K Svanberg AS. Satisfaction with childbirth services provided in public health facilities: results from a cross- sectional survey among postnatal women in Chhattisgarh, India. Glob Health Action. (2017) 10(1):1386932. 10.1080/16549716.2017.1386932 29087240 PMC5678347

[12] Panth A. Maternal satisfaction on delivery service among postnatal mothers in a government hospital, Mid-Western Nepal. Obstet Gynecol Int. (2018) 2018:4530161. 10.1155/2018/4530161 30034472 PMC6035828

[13] Sayed W Abd El Aal DE Mohammed H Abbas A Zahran K. Maternal satisfaction with delivery services at tertiary university hospital in upper Egypt, is it actually satisfying?. Int J Reprod Contracept Obstet Gynecol. (2018) 7(7):2547–52. 10.18203/2320-1770.ijrcog20182859

[14] Adjei KK Kikuchi K Owusu-Agyei S Enuameh Y Shibanuma A Ansah EK Women ’ s overall satisfaction with health facility delivery services in Ghana: a mixed-methods study. Trop Med Health. (2019) 47:1–9. 10.1186/s41182-018-0133-6 31320830 PMC6612170

[15] Mocumbi S Högberg U Lampa E Sacoor C Valá A Bergström A Mothers ’ satisfaction with care during facility-based childbirth: a cross-sectional survey in southern Mozambique. BMC Pregnancy Childbirth. (2019) 19:1–14. 10.1186/s12884-019-2449-6 31426758 PMC6701029

[16] Demis A Nigatu R Assefa D Gedefaw G. Women ’ s satisfaction with intrapartum care in St Paul ’ s hospital millennium medical college Addis Ababa Ethiopia: a cross sectional study. BMC Pregnancy Childbirth. (2017) 17:1–8. 10.1186/s12884-017-1428-z 28754136 PMC5534094

[17] Zemenu Yohannes Kassa TA. Maternal satisfaction and associated factors on delivery care service in gynecology & obstetrics maternal satisfaction and associated factors on delivery care service in hawassa city public hospitals, South Ethiopia. Gynecol Obstet (Sunnyvale). (2019) 8(473):2161–0932. 10.4172/2161-0932.1000473

[18] Gashaye KT Tsegaye AT Shiferaw G Worku AG Abebe SM. Client satisfaction with existing labor and delivery care and associated factors among mothers who gave birth in university of Gondar teaching hospital; Northwest Ethiopia: institution based cross-sectional study. PLoS ONE. (2019b) 14(2):1–15. 10.1371/journal.pone.0210693 PMC6364872 30726297

[19] Demis A Nigatu R Assefa D Gedefaw G. Maternal satisfaction with intrapartum nursing care and its associated factors among mothers who gave birth in public hospitals of North Wollo Zone, Northeast Ethiopia: institution-based cross-sectional study. J Pregnancy. (2020) 2020:8279372. 10.1155/2020/8279372 32395345 PMC7201724

[20] Deriba BS. Maternal Satisfaction and Factors Associated with Institutional Delivery Care in Central Ethiopia: a Mixed Study (2021).

[21] Silesh M Lemma T. Maternal satisfaction with intrapartum care and associated factors among postpartum women at public hospitals of North Shewa Zone Ethiopia. PloS one. (2021) 16(12):e0260710. 10.1371/journal.pone.0260710 34852019 PMC8635333

[22] Berhe H. Status of caring, respectful and compassionate health care practice in tigrai regional state: patients ’ perspective. Int J Caring Sci. (2017) 10(3):1118–28.

[23] Abebe A Menta AA. Nutrition and dietetic practice mothers ’ satisfaction with institutional delivery service and associated factors among women attending hospitals in. Pregnancy Child Birth. (2018) 2(2):1–12.

[24] Asres GD. Satisfaction and associated factors among mothers delivered at asrade zewude memorial primary hospital, bure, west gojjam, amhara, Ethiopia: a cross sectional study. Primary Health Care. (2018) 08(02):293. 10.4172/2167-1079.1000293

[25] Bishaw KA Temesgen H Amha H Desta M Bazezew Y Ayenew T A systematic review and meta-analysis of women’s satisfaction with skilled delivery care and the associated factors in Ethiopia. SAGE Open Med. (2022) 10:205031212110682. 10.1177/20503121211068249 PMC8785278 35083043

[26] Ngowi AF Kamazima SR Kibusi S Gesase A Bali T. ‘Women’s determinant factors for preferred place of delivery in Dodoma region Tanzania: a cross sectional study’. Reprod Health. (2017) 14(1):1–8. 10.1186/s12978-017-0373-7 28877749 PMC5588730

[27] Delibo D Damena M Gobena T Balcha B. Status of home delivery and its associated factors among women who gave birth within the last 12 months in east badawacho district, hadiya zone, southern Ethiopia. BioMed Res Int. (2020) 2020:8. 10.1155/2020/4916421 PMC7453228 32923481

[28] Kifle MM Kesete HF Gaim HT Angosom GS Araya MB. Health facility or home delivery? Factors influencing the choice of delivery place among mothers living in rural communities of Eritrea. J Health Popul Nutr. (2018) 37:1–15. 10.1186/s41043-018-0153-1 30348219 PMC6196428

[29] Makuka GJ Sango MM Mashambo AE Mashambo AE Msuya SE Mtweve SP. Clients’ perspectives on quality of delivery services in a rural setting in Tanzania: findings from a qualitative action-oriented research. Int J Matern Child Health AIDS (IJMA). (2017) 6(1):60–8. 10.21106/ijma.191 PMC5547226 28798894

[30] Dar B. Compassionate and respectful maternity care during facility based child birth and women ’ s intent to use maternity service in Bahir Dar, Ethiopia. BMC Pregnancy Childbirth. (2018) 18:1–9. 10.1186/s12884-017-1633-9 29986659 PMC6038196

[31] Federal Democratic Republic of Ethiopia Ministry of Health. ‘Health Sector Transformation Plan II 2020/21-2024/25 (2013EFY-2017EFY)’, 25(February) (2021).

[32] Demographic M Survey H. *Ethiopia* (2019).

[33] Central Statistical Agency. 2007 Population and housing census of Ethiopia (2007).

[34] Reta T Tadesse L. Satisfaction with childbirth services given in public health facilities: a cross-sectional survey in Ethiopia. J Women’s Health Care. (2021) 10(540):2167–0420. 10.35248/2167-0420.21.10.540

[35] Babure K. Maternal satisfaction and associated factors towards delivery service among mothers sectional study design. J Women’s Health Care. (2020) 9(489):2167–0420. 10.35248/2167-0420.20.9.489

[36] Hailemariam S Genetu A Sahile E. Mother’s satisfaction towards childbirth care at public health centers in Bench-Maji Zone, Ethiopia: a facility-based cross-sectional study. Int J Reprod Med. (2020) 2020:1–7. 10.1155/2020/6746459 PMC7354652 32695806

[37] Gejea T Abadiga M Hasen T. Maternal satisfaction with delivery services of government hospitals in Ambo Town, West Shoa Zone, Oromia Region, Ethiopia, 2020. Patient Prefer Adherence. (2020) 14:1225–35. 10.2147/PPA.S251635 32801653 PMC7383021

[38] Wolka S Assegid S Tantu T Gunta M Duko B. Determinants of maternal satisfaction with existing delivery care at wolaita Sodo university teaching and referral. BioMed Res Int. (2020) 2020:6403123. 10.1155/2020/6403123 33029519 PMC7533011

[39] Edaso AU Teshome GS. ‘Mothers ’ satisfaction with delivery services and associated factors at health institutions in west arsi, oromia regional state, Ethiopia. MOJ Womens Health. (2019) 8(1):110–9. 10.15406/mojwh.2019.08.00222

[40] Bulto GA Demissie DB Tasu TL Demisse GA. Mother’s satisfaction with the existing labor and delivery care services at public health facilities in West Shewa zone, oromia region, Ethiopia. BMC Pregnancy Childbirth. (2020) 20(1):1–12. 10.1186/s12884-020-02998-6 PMC7236095 32429878

[41] Assefa B. Maternal satisfaction with delivery services of public health centers in Addis Ababa, Ethiopia, 2017 GC By:—Blen Assefa (Bsc) Maternal satisfaction with delivery services ofpublic healt’ (2017).

[42] Debela AB Mekuria M Kolola T Bala ET Deriba BS. Maternal satisfaction and factors associated with institutional delivery care in central Ethiopia: a mixed study. Patient Prefer Adherence. (2021) 15:387–98. 10.2147/PPA.S297662 33642855 PMC7903958

[43] Gudayu TW Araya BM. Outcomes among mothers who gave birth in the health facility: does birth preparedness and complication readiness have a role? Obstet Gynecol Int. (2019) 2019:6. 10.1155/2019/5147853 PMC6515023 31182963

[44] Getenet AB Teji Roba K Seyoum Endale B Mersha Mamo A Darghawth R. Women’s satisfaction with intrapartum care and its predictors at harar hospitals, eastern Ethiopia: a cross-sectional study. Nurs Res Rev. (2018) 9:1–11. 10.2147/nrr.s176297

[45] Getachew T Eyeberu A Dheresa M. Maternal satisfaction on delivery care services and associated factors at public hospitals in eastern Ethiopia maternal satisfaction on delivery care services and associated factors at public hospitals in eastern Ethiopia. Int Health. (2022) 15:189–97. 10.1093/inthealth/ihac038 PMC9977211 35668629

[46] Shepherd A Cheyne H. The frequency and reasons for vaginal examinations in labour. Women Birth. (2013) 26(1):49–54. 10.1016/j.wombi.2012.02.001 22397830

[47] Zampas C Amin A O'Hanlon L Bjerregaard A Mehrtash H Khosla R Operationalizing a human rights-based approach to address mistreatment against women during childbirth. Health Hum Rights. (2020) 22(1):251–64. 32669805 PMC7348458

